# Paving the Way to Understand Autoantibody-Mediated Epilepsy on the Molecular Level

**DOI:** 10.3389/fneur.2015.00149

**Published:** 2015-07-06

**Authors:** Guiscard Seebohm, Ilaria Piccini, Nathalie Strutz-Seebohm

**Affiliations:** ^1^Receptor Structure and Function Group, Department of Cardiovascular Medicine, Institute for Genetics of Heart Diseases (IfGH), University Hospital Münster, Münster, Germany

**Keywords:** limbic encephalitis, glutamate receptor, ion channel, epitope identification, modeling

## Abstract

Correct function of neuronal networks is enabled by a delicate interplay among neurons communicating with each other. One of the keys is the communication at chemical synapses where neurotransmitters like glutamate, GABA, and glycine enable signal transfer over the synaptic cleft. Thereby, the neurotransmitters are released from the presynapse and bind as ligands to specific receptors at the postsynaptic side to allow for modulation of the postsynaptic membrane potentials. The postsynaptic electrical signal, which is highly modulated by voltage-gated ion channels, spreads over the dendritic tree and is thus integrated to allow for generation of action potentials at the axon hillock. This concert of receptors and voltage-gated ion channels depends on correct function of all its components. Misfunction of receptors and/or voltage-gated potassium channels (VGKC) leads to diverse adverse effects in patients. Such malfunctions can be the result of inherited genetic alterations or pharmacological side effects by drugs. Recently, autoantibodies targeting receptor or channel complexes like NMDAR, AMPAR, GABA-receptors, glycine receptors, LGI1 or CASPR2 (previously termed as VGKC-complex antibodies) have been discovered. The presence of specific autoantibodies against these targets associates with severe forms of antibody-mediated encephalitis. Understanding the molecular details of autoantibody actions on receptor and VGKC complexes is highly desirable and may open the path to develop specific therapies to treat humoral autoimmune encephalitis. Here, we summarize the current knowledge and discuss technical approaches to fill the gap of knowledge. These techniques include electrophysiology, biochemical approaches for epitope mapping, and *in silico* modeling to simulate molecular interactions between autoantibody and its molecular target.

The neuronal activity is the basis for normal brain function. Altered activity can result in neurological diseases like epilepsy, insomnia, hallucinations, schizophrenia, different forms of headache or autism, and even death (1). Vital for normal brain function is the correct formation of action potentials in neurons and chemo-electrical communication among neuronal cells. Molecular actuators include voltage-gated potassium channels (VGKC) and ligand-gated ionotropic receptors like the excitatory glutamate or the inhibitory glycine receptors. Impaired function of these molecular key players can result from inherited mutations or as side effect of pharmacologically active compounds. Both, activation and inhibition of molecular targets may lead to disharmonies in the concert of brain function. Recently, a new entity has been added to the modulators of neuronal key players that alter their function: autoantibodies. These penetrate the blood–brain barrier or are intrathecally synthesized to bind to epitopes of neuronal protein complexes to alter their function (2). Depending on their glutamate receptor target, these were named as *N*-methyl-d-aspartate receptor (NMDAR) encephalitis (2) or α-amino-3-hydroxy-5-methyl-4-isoxazolepropionic acid receptor (AMPAR) encephalitis (autoantibodies targeting NMDAR or AMPAR) (3). Glutamate receptors are expressed in various types of benign and malignant neoplasms, play a role as growth factors and are important for cancer development and progression (4). Expression of glutamate receptors in tumor cells triggers an anti-tumor immune response, which can suppress tumor growth and symptoms (5, 6). This tumor immune response can break the immune tolerance and different glutamate receptor autoantibodies can attack the neuronal tissue (paraneoplastic syndrome).

Another form of autoantibody encephalitis is the result of antibody binding to LGI1 or CASPR2 (7–9). Increasing evidence suggests that interference with LGI1 binding to ADAM22 modulates postsynaptic AMPAR and possibly presynaptic VGKC (10). These autoantibody encephalitides with plasma membrane expressed antigens may primarily lead to impaired neurotransmission to cause the specific disease state. The membrane localized autoantibody targets are intracellular proteins involved in transcription, post-transcriptional RNA regulation, and cytoplasmic proteins with various cellular functions. These intracellular target-associated autoantibody encephalitides are associated with CD8 T-cell activation and neuronal apoptosis. The clinical manifestations of these pathophysiological events include forms of limbic encephalitis with epileptic neuronal activity of the mediotemporal lobe associated with memory impairment, behavioral aberrations, emotional, and cognitional malfunction. The latter clinical symptoms are only weakly understood. The number of cases of neuronal inflammatory autoimmune diseases is supposedly underestimated.

In recent years, the knowledge about autoantibodies in limbic encephalitis has increased. In this article, we concentrate on the plasma membrane antigens to understand the pathophysiological mechanisms associated with these forms of autoantibody encephalitis. Autoantibodies targeting NMDAR have been reported 8 years ago (2). The autoantibody epitope is located on GluN1 (1). Retrospective diagnoses have identified a larger number of cases as autoantibody-NMDAR encephalitis (11). These findings suggest that anti-NMDAR encephalitis is more common than previously thought. This knowledge is valuable as the speed of diagnosis of autoantibody-NMDAR encephalitis is key to the success of clinical recovery (1, 12, 13): the earlier the autoantibody-NMDAR encephalitis is diagnosed and immunomodulation therapy is started, the better are the chances for complete recovery of the patient. What is the molecular–cellular action of autoantibody-NMDAR? First indications came from application of NMDAR-autoantibodies in hippocampal neurons (14, 15). Zhang et al. and Panaguma et al. showed that NMDAR-autoantibodies weakened long-term potentiation (LTP) in hippocampal neurons (15, 16). This shows that LTP in synaptic transmission critically depending on NMDAR is substantially weakened. LTP is enabled by intact endocytic cycling via a RAB5–RAB11 pathway in hippocampal neurons (17). In an elaborate study, Hughes et al. showed that NMDAR-autoantibodies can cluster NMDA-receptors to induce endocytosis (14). Application of antibody fragments (FAB) was not effective to induce endocytosis. When these FABs were clustered by anti-FABs endocytosis was reinduced. The reduction of NMDAR-plasma membrane density was verified in rodent and human hippocampi (14, 18). Using a series of mutant receptor proteins and mass spectrometry analyses, Gleichman et al. showed that residue N368 in GluN1 is glycosylated and modulates electrophysiological effects of NMDAR-autoantibody binding (19). Binding of NMDAR-autoantibodies results in prolonged openings of NMDAR leading to a gain-of-function. However, the reported gain-of-function by NMDAR-autoantibody binding was later challenged and may not be indispensable for the reduced NMDAR-plasma membrane density (18). Post-endocytic trafficking of NMDARs may not be affected by acute NMDAR-autoantibody binding (18). Interestingly, the region around this glycosylation site controls recognition by NMDAR-autoantibodies, but glycosylation itself is neither necessary nor sufficient for NMDAR-autoantibody immunoreactivity (19). Summarizing, NMDAR-autoantibodies bind to their epitope on the receptor, modulate protein complex conformation to induce clustering and subsequent endocytosis. However, the exact physical NMDAR-autoantibody binding site remains to be proven.

Six years ago, Lai et al. reported on AMPAR-autoantibody limbic encephalitis (3). Using immunoprecipitation and mass spectrometry anti-GluA1/GluA2-autoantibodies were identified. Heterologously expressed GluA1/GluA2 receptors and rodent cultivated hippocampal neurons were used to show immunolocalization and increased anti-GluA1/GluA2-autoantibody-mediated endocytosis of GluA1/GluA2 complexes (3, 20). ELISA tests were used to quantify anti-GluA1/GluA2-autoantibodies in cancer patients (3). The anti-GluA1/GluA2-autoantibody positive patients often had tumors and supposedly the anti-GluA1/GluA2-autoantibodies were directed against these cancers and had secondary adverse neuronal effects. Unluckily, these patients tended to suffer from relapse (3). The knowledge about the molecular and cellular effects of these antibodies has been insufficient.

The current knowledge on the molecular and cellular level of autoantibody binding to plasma membrane proteins is far from complete. Several questions remain to be addressed. Hereafter, we will suggest experimental approaches to stimulate research regarding pressing questions in this clinically important area.

Why are autoantibodies directed against glutamate receptors, leucine-rich, glioma inactivated 1 protein, and CASPR2 but not against Kv1 α-subunits reported?

Membrane protein complexes consist of several protein components. Some of them are membrane integral with extracellular domains of various size and some are intracellular (Figure 1). Large protein parts of glutamate receptors, LGI1 and CASPR2, are exposed to the extracellular space. The surfaces of these protein fractions represent potential epitopes against which (auto-) antibodies can be directed. On the contrary, a potassium channel α-subunit extends to the extracellular space with small surfaces presenting a relatively small area and thus restricted epitopes. Consistently, (auto-) antibody binding to the large extracellular domains of glutamate receptors seems more probable than to Kv1 α-subunit. This oversimplifying hypothesis is based on the idea that the autoantibodies bind exclusively to extracellularly exposed protein regions. This hypothesis is supported by the finding that AMPAR, NMDAR, leucine-rich, glioma inactivated 1 protein, and CASPR2 but not Kv channel autoantibodies have been reported. Pathophysiologically relevant autoantibodies against intracellular epitopes exist as well. These antibodies have to penetrate cellular membranes to reach their epitopes and are therefore supposedly less common.

**Figure 1:**
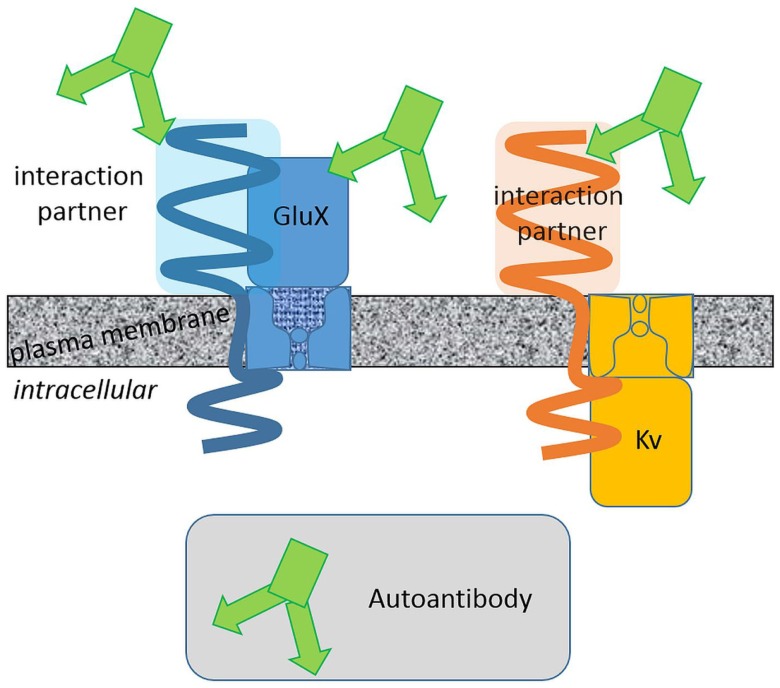
**Extracellular surfaces determine the autoantibody interaction sites**. Autoantibodies associated with limbic encephalitis target different glutamate receptor and Kv channel complexes. Integral proteins with large extracellular domains present larger surfaces potentially suited as autoantibody epitopes. Binding of autoantibodies against these proteins with large extracellular domains is more likely.

The extracellular epitope idea is helpful to design experimental approaches to identify autoantibody epitopes. For none of the plasma membrane autoantibody targets, the exact epitope was determined. However, the knowledge of the exact epitope(s) is/are potentially highly valuable to further understand the autoantibody action. Potentially, the pathophysiological autoantibody–epitope interaction can be disrupted by a synthetic peptide of the epitope, a peptidomimetic or even a small molecule inhibitor. These compounds could be used to bind to the autoantibodies and outcompete the natural epitopes on the host membrane proteins as a therapeutic option. Such a specific therapy may be favorable and reduce the undesired side effects intrinsic to global immunosuppressant therapy. Identification of an autoantibody epitope is technically not trivial. On the other hand, a series of technical approaches to identify interaction sites between proteins or proteins with small molecule modulators exists (21). A classical approach is to generate chimeras between two closely related proteins only one of which can bind the ligand. By testing direct binding or testing the functional effect upon binding and progressively reducing the size of the region of interest, the putative binding region can be narrowed down. Ideally, gain-of-function and loss-of-function approaches are adopted in parallel. When a smaller region is identified, a point mutational approach in the respective region can identify molecular prerequisites for the interaction of binding partners. This approach largely leaves the structural determinants of the interaction site intact, a fact not accounted for by using fractional peptide-based assays, which are favorable for misfolded proteins (Figure 2). The design of (point-) mutants can be aided by structural modeling approaches. Crystal structures of different glutamate receptors and Kv channels allow for high-resolution 3D homology modeling. These models can be used to identify the surface exposed residues. These residues are potentially involved in the formation of autoantibody epitopes. Mutagenesis of only these residues reduces the experimental effort. Further, the transmembrane and intracellular regions can be excluded as autoantibody epitopes based on the extracellular epitope idea (Figures 1 and 2). The interaction of the autoantibody with its specific target can be assayed by several approaches. A classical approach would be expression of wt and mutated membrane protein complexes in *Xenopus laevis* oocytes or a cell line like HEK293 cells and functional analyses upon subsequent autoantibody application. Clearly, a prerequisite of this approach is that the autoantibody causes clear functional effects on its target. A disadvantage is that the effect upon binding but not the binding itself is detected. Alternatively, direct binding may be detected. Similar to the electrophysiology-based approach, mutant targets are heterologously expressed. The autoantibody is applied. Subsequently, the autoantibody is cross-linked by bi-functional cross-linkers. As a result, the antibody is cross-linked to the target and can be detected by subsequent target protein purification and western blotting or mass spectrometry. When binding of the antibody to the target is disrupted by a specific mutation the antibody will no longer be cross-linked and co-precipitated with the target protein. In patient sera, more than one autoantibody binding different epitopes on the same target can exist. In this case, the epitopes may cluster in different regions of the target.

**Figure 2:**
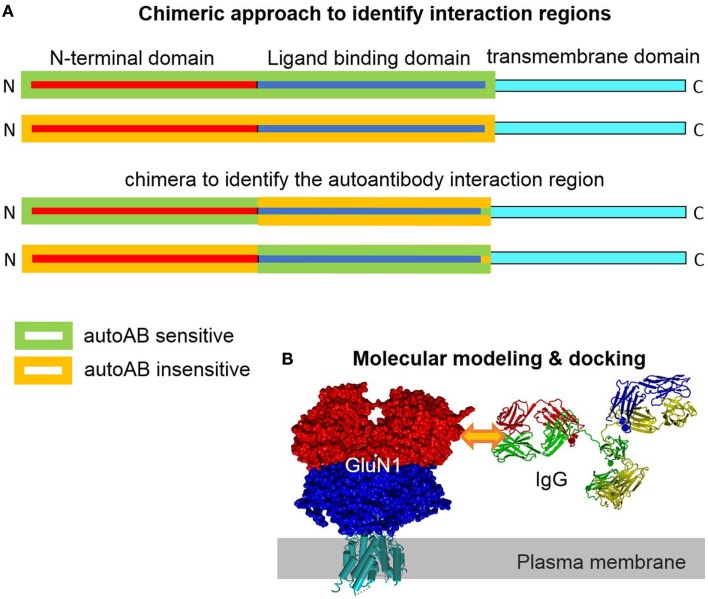
**Chimeric and homology modeling/docking approach to identify and simulate autoantibody-target protein interaction**. **(A)** Construction of a chimera of closely related target proteins but with different autoantibody binding affinities can help to identify epitope regions in the target protein. Complementary gain-of-function and loss-of-functional approaches provide evidence for the location of the interaction region. **(B)** Models of the target (GluN1) and the specific IgG can be generated based on solved highly homologous crystal structural coordinates. Docking of the interaction partner in the region of the experimentally determined epitope site allows for analysis of structural effects *in silico*.

The three dimensional structures of several membrane proteins, including GluN, GluA, and Kv channels, have been solved (22–24). These high-resolution structures represent very good templates for homology modeling of the autoantibody targets. Standard homology modeling will allow for generation and localizations of the identified epitopes. If the sequence of the IgG-autoantibody is known, its structure can be modeled as well. High-resolution template IgG structures like 1IGT.pdb (25) are perfectly suited for homology modeling. Molecular docking of this antibody model to one or two membrane protein targets will allow to study the molecular details *in silico* (Figure 2B). If more than one epitope is targeted by antibodies, the epitopes on the structural models can show distinct localizations. Dissection of overlapping epitopes, however, may be difficult. This new structural knowledge should be useful to develop drug candidates and therapeutic approaches.

## Conflict of Interest Statement

The authors declare that the research was conducted in the absence of any commercial or financial relationships that could be construed as a potential conflict of interest.
